# Case report: Electroacupuncture for acute pain flare-up of knee osteoarthritis

**DOI:** 10.3389/fneur.2022.1026441

**Published:** 2022-10-25

**Authors:** Hai Huang, Yongying Liang, Dapeng Han, Xiaoyan Chen, Lianbo Xiao, Hongyan Wu

**Affiliations:** ^1^Geriatrics Department, Shenzhen Hospital, Shanghai University of Traditional Chinese Medicine, Shenzhen, China; ^2^Graduate School, Shanghai University of Traditional Chinese Medicine, Shanghai, China; ^3^Joint Surgical Department, Guanghua Hospital, Shanghai University of Traditional Chinese Medicine, Shanghai, China

**Keywords:** electroacupuncture, acute pain flare-up, knee, osteoarthritis, case report

## Abstract

Acute pain flare-up of knee osteoarthritis (KOA) is a common disease in orthopedics and is mainly treated with analgesic drugs. Patients usually refuse to take western medicines orally owing to gastrointestinal side effects or unsatisfactory treatment results. We report the case of a 69-year-old woman who had an acute pain flare-up of right KOA induced by long-distance walking. As the patient refused medication, we used electroacupuncture (EA) to relieve her symptoms. EA with a 2-Hz frequency and a 1–2-mA intensity had an analgesic effect on the acute pain flare-up of KOA. After 12 weeks of EA intervention, the bone marrow edema-like lesions (BMLs) improved significantly, as depicted on magnetic resonance imaging of the knee joint. However, more powerful evidence is needed to understand the mechanism of the EA technique that alleviates BMLs of KOA.

## Introduction

With the aging global population, the prevalence of knee osteoarthritis (KOA) is increasing (1). The primary complaints of patients with KOA are pain and poor joint mobility, which seriously affect their daily life and are the most common reasons for doctor visits. Pathological changes in patients with KOA mainly include cartilage damage and hyperosteogeny around the joints, which then irritates the surrounding soft tissues, resulting in soft tissue hypertrophy, inflammatory edema, and blood stasis.

An X-ray image of the knee joint is the most commonly used and popular method to diagnose KOA at a clinic. This facilitates the grading of KOA using the Kellgren–Lawrence (K–L) grading scale, which was made possible by improvements in X-ray imaging. Previous magnetic resonance imaging (MRI)-based studies have shown that the pain is related to many factors, including joint effusion, bone marrow edema, and osteoarthritis (2–4). Bone marrow lesions of knee joints in patients with osteoarthritis (OA-BML) are important clinical entities, which can explain progressive pain (5), decreased quality of life, and impaired function. MRIs of bone marrow edema-like lesions (BMLs) showed subchondral bone areas with hyperintense marrow signals on T2-weighted imaging and are closely related to the pain, subchondral bone cyst formation, and the progression of KOA (6, 7).

Multiple activity-related, psychosocial, and environmental factors easily trigger acute KOA flare-ups (8). Acupuncture is an effective non-drug strategy for treating acute and chronic pain (9, 10). Pain, stiffness, or swelling are common symptoms of acute flare-ups in patients with KOA (11). Bartholdy et al. (12) showed that a predefined and standardized “rescue” exercise may be beneficial in patients with exacerbated KOA symptoms. However, few clinical guidelines cover evidence-based management strategies of non-drug therapy for reducing the impact of KOA flare-ups. Acupuncture has been widely used as a non-drug therapy for neurological pain and arthropathy (13). Here, we report the case of a patient with an acute KOA flare-up, which was treated successfully using electroacupuncture (EA).

## Case description

A 69-year-old woman presented at the acupuncture clinic on 30 June 2018. The patient was pushed in a wheelchair. Her primary complaints were knee pain and inability to walk. Just 1 month previously, the patient had completed a long-distance walk of approximately 445 km. This had induced an acute flare-up of knee pain. For almost 1 month, she anticipated that she could recover unaided and, hence, did not seek treatment, or self-administer Chinese or western medication; however, she was still experiencing pain.

**Figure 1:**
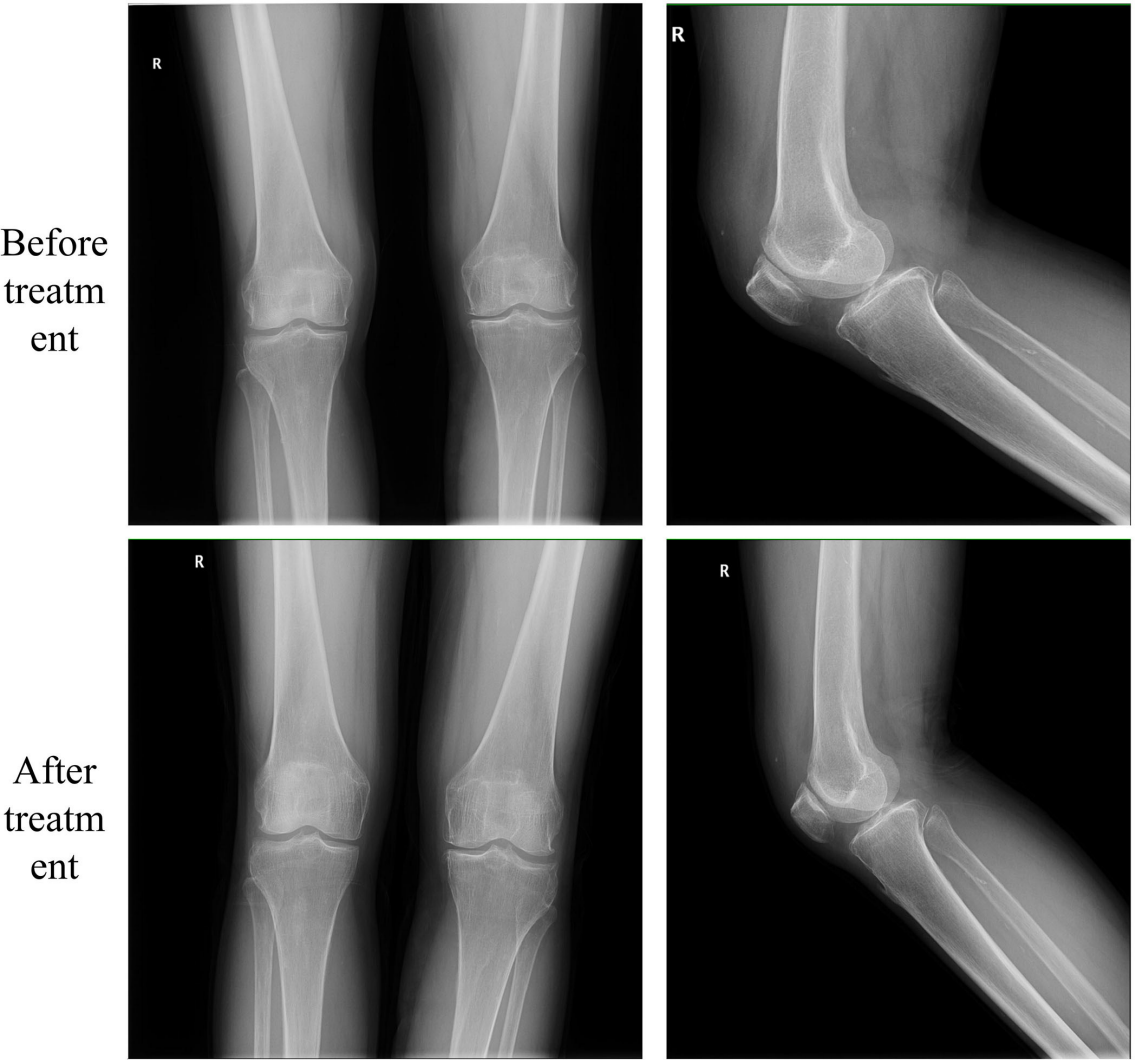
X-ray before and after treatment. X-rays of the knee before treatment were suggestive of knee osteoarthritis (Grade III on the K–L grading). K–L, Kellgren–Lawrence.

**Figure 2:**
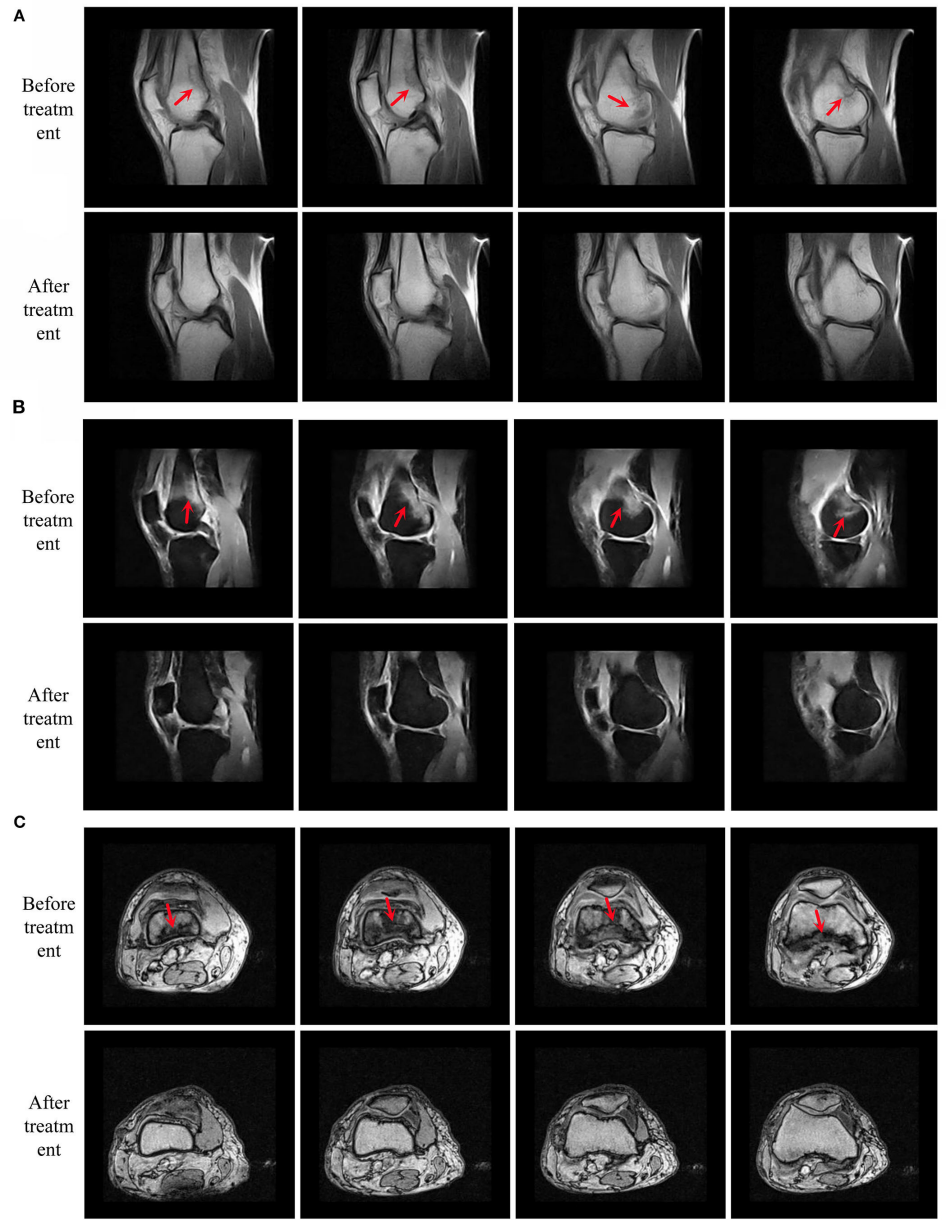
MRIs compared before and after treatment. Present four consecutive magnetic resonance images from left to right. **(A)** Sagittal conventional sequence. **(B)** Sagittal fat-suppressed sequence and **(C)** axial sequence. Serious bone marrow edema-like lesions (the red arrow). MRI, magnetic resonance imaging.

The patient laid flat on the treatment bed and the doctor observed redness and swelling, without obvious deformation of the right knee joint during the examination; however, joint tenderness and a pronounced medial side were apparent. The range of motion of her knee joint was 40°, and her Western Ontario and McMaster Universities Osteoarthritis Index (WOMAC) score was 92 points, including 25, 11, and 56 points for pain, joint stiffness, and physical function, respectively. Her Lequesne index and visual analogue scale (VAS) scores were 11 and 9 points, respectively. Immediately prior to treatment, X-ray (Figure 1) and MRI examination (Figure 2) of the right knee joint were performed.

According to the symptoms, physical examination, and radiograph, we diagnosed the patient with KOA (14), with an acute pain flare-up in the right knee joint. However, X-ray showed that KOA was more serious in the left knee. As she continued to refuse medication, we used EA to relieve her symptoms.

**Figure 3:**
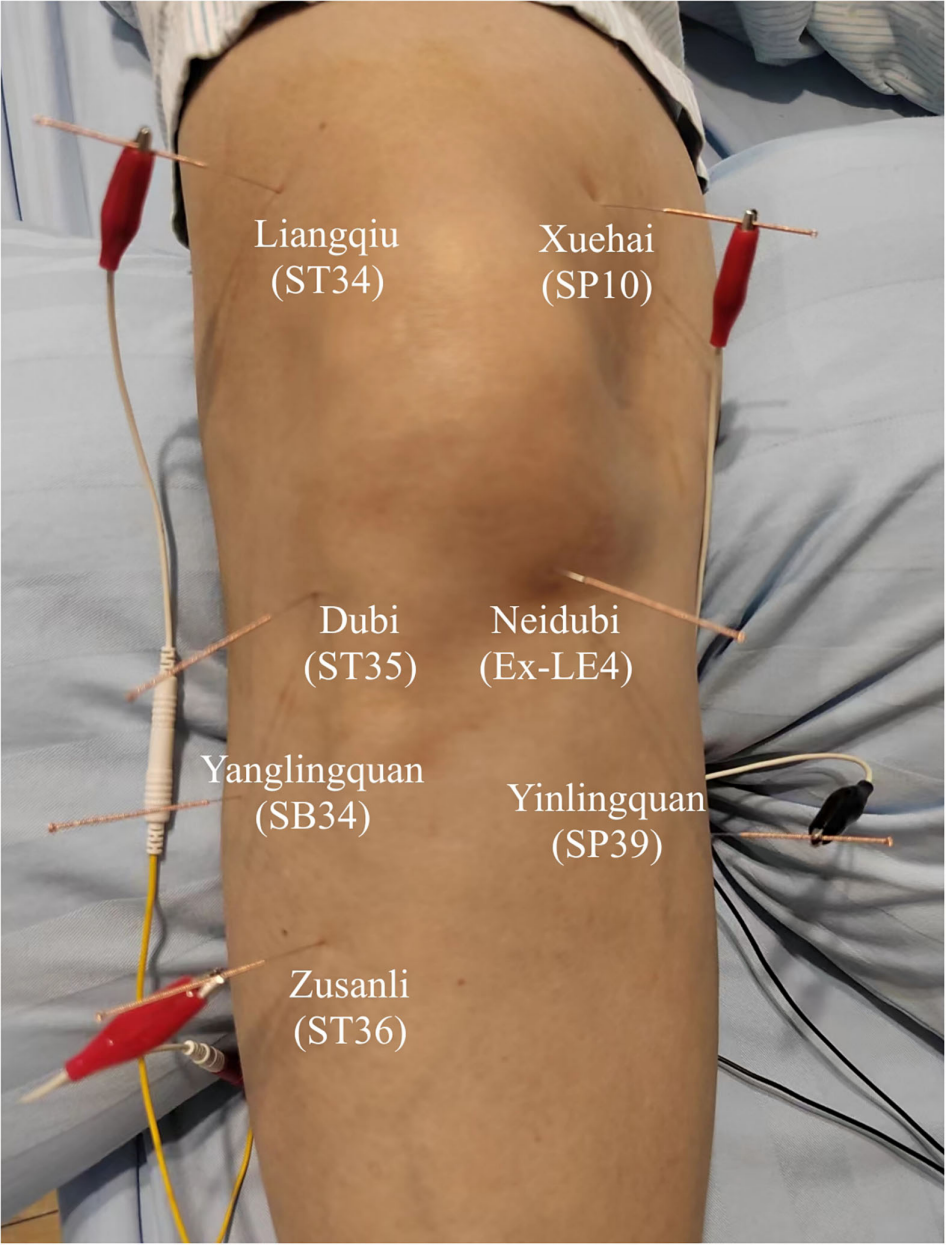
Locations of acupoints.

According to the principles of nearby acupoint selection and the synergistic effect of *yin* and *yang* relative acupoints of the knee joint, we selected *Liangqiu* (ST34), *Xuehai* (SP10), *Neidubi* (Ex-LE4), *Dubi* (ST35), *Yanglingquan* (GB34), *Yinlingquan* (SP9), and *Zusanli* (ST36) (Figure 3).

The operating procedure was as follows: The patient laid flat on the treatment bed, and the acupuncturist stood on the right side of the patient to locate the acupoints. After skin disinfection, the acupuncturist inserted the acupuncture needle (0.30 ^*^ 40 mm, Suzhou Medical Appliance Factory, Suzhou, China) to a 30-mm depth under the skin. After the patient experienced a sense of *deqi*, the acupuncturist connected two pairs of EA connectors to the needle handles of ST34–ST36 and SP10–SP9. The waveform of the electrical stimulation (SDZ-V electroacupuncture apparatus, Suzhou Medical Appliance Factory) was set to continuous wave, with a 2-Hz frequency, 1–2-mA intensity, and 30-min duration. Treatment was repeated three times per week for 12 weeks (36 treatments in total). EA was performed by the same experienced acupuncturist who was registered in China. During the treatment, no medications were used, and no abnormal acupuncture conditions (such as pain, subcutaneous hemorrhage, needle bending, broken needle, or needle stagnation) were observed.

**Figure 4:**
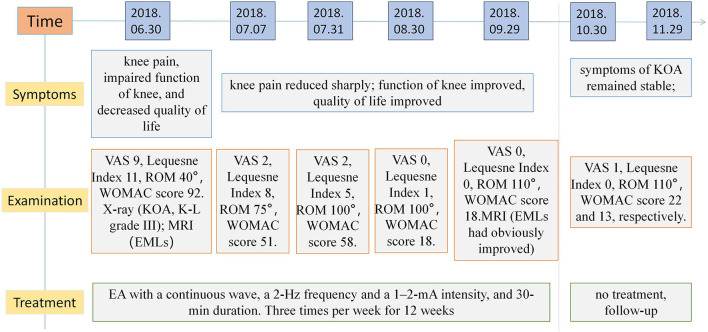
Study timeline. VAS, visual analogue scale; WOMAC, The Western Ontario and McMaster Universities Osteoarthritis Index; ROM, range of motion; MRI, magnetic resonance imaging; KOA, knee osteoarthritis; EMLs, bone marrow edema-like lesions; EA, electroacupuncture.

As shown in Table 1 and Figure 4, at 30 min after treatment, the pain in the right knee joint of the patient was relieved, and the VAS score was 5. Subsequently, the VAS score was 2 after 1 and 4 weeks of treatment and further decreased to 0 after 8 and 12 weeks of treatment. The patient was followed up at the clinic after treatment, and her VAS score was 1. Her total WOMAC score decreased as treatment progressed, and the pain score remained low after the treatment was completed. The Lequesne index indicates the severity and activity index of KOA, and the score gradually decreased as the acupuncture treatment progressed. The joint range of joint motion increased as the pain was relieved. After 12 weeks of EA treatment, MRI showed that the area of bone marrow edema-like lesions had decreased.

**Table 1:** Clinical assessments for pain and knee joint function at each time points.

**Clinical assessments**		**Before treatment**	**30 minutes after first treatment**	**1 week after treatment**	**4 weeks after treatment**	**8 weeks after treatment**	**12 weeks after treatment**	**4 weeks follow up**	**8 weeks follow up**
VAS score		9	5	2	2	0	0	1	1
Lequesne index		11	–	8	5	1	0	0	0
ROM (°)		40	–	75	100	100	110	110	110
WOMAC Score	Pain	25	–	10	10	2	4	5	3
	Stiffness	11	–	10	9	0	0	0	0
	Physical function	56	–	31	39	16	14	17	10
	Aggregate score	92	–	51	58	18	18	22	13

There was no obvious pain in the knee joint at follow-up, and its range of motion had reached 100–110°. Over the following 2 years, the knee pain score was maintained at 1–2 points, with no negative influence on daily life, as determined by telephonic follow-up.

## Discussion

This case greatly impressed the authors. The patient was unable to walk at the first visit and was pushed in a wheelchair. She was able to walk with crutches after the second treatment and walked unaided after the third. Compared with other treatments (such as drug therapy, weight loss, and joint function exercises) (15), EA has fewer side effects and higher safety and is readily accepted by patients in China. After three treatments, the patient, who had not previously undergone acupuncture, was satisfied with the therapeutic effect and referred to it as “the gift of acupuncture.”

Long-term use of non-steroidal anti-inflammatory drugs has adverse effects on renal function and may cause gastrointestinal bleeding (16). Many recent studies havesuggested the use of non-drug therapy, and traditional Chinese medicine may be a viable alternative for patients with KOA. Acupuncture has a long history in osteoarthritis treatment (17). The American College of Rheumatology and the International Osteoarthritis Research Association also recommend acupuncture as a symptomatic relief treatment for patients with KOA who are unwilling to undergo total knee arthroplasty (18, 19). Commonly used acupoints for KOA are ST34, ST36, GB34, and SP9. These acupuncture points are very close to the knee joint, and most are located on the muscles attached to the tibia/fibula or patella (20). KOA is a complex chronic pain disease, partly due to its nociceptive and neurological mechanisms. It is usually accompanied by neuroplasticity and central nervous system pain sensitization (21–23). This case was of a patient with an acute pain flare-up of right KOA induced by long-distance walking. The patient showed obvious pain, and the symptoms had not disappeared after a month. Previous researches have shown that warming acupuncture and EA may be optimal acupuncture methods for treating KOA (24, 25). In this study, EA significantly reduced the VAS and WOMAC scores of our patient. Modern medical research shows that endogenous opioid peptides in the central nervous system play an essential role in mediating the analgesic effect of EA (26). Acupuncture encourages the release of endorphins or other monoamines through afferent nerve stimulation of the spinal cord, thus, blocking pain signals and producing analgesic effects (27).

The possible mechanism of acupuncture for improvement in KOA remains unclear. Ruan et al. (28) showed that EA alleviated the inflammation and histological changes in KOA rabbits by inhibiting the toll-like receptor-mediated innate synovial immune response. Li et al. (29) showed that acupuncture treatment may inhibit the MCP1/CCR2 axis and downregulate the inflaming factor and nerve growth factor in the cartilage and synovial tissue. However, only a few studies have examined acupuncture-associated improvement in local BMLs of the knee joint. Xu et al. (30) showed that MBLs were associated with the progression of articular cartilage loss and fluctuation of the pain in KOA. After EA intervention, the BMLs shown in the MRI of the knee joint of our patient were clearly improved. However, although our case report suggests that acupuncture could potentially alleviate BMLs of the knee joint to relieve symptoms, further placebo-controlled studies with larger sample sizes are required to verify this conjecture.

## Conclusion

In this study, we present a case of acute pain flare-up of KOA that was successfully treated with EA. EA with a 2-Hz frequency and 1–2-mA intensity had an analgesic effect and was beneficial for the alleviation of symptoms. The potential mechanism of EA on acute pain flare-up associated with KOA is to reduce BMLs.

## Data availability statement

The original contributions presented in the study are included in the article/supplementary material, further inquiries can be directed to the corresponding authors.

## Ethics statement

The studies involving human participants were reviewed and approved by the Institutional Review Board of Guanghua Hospital, Shanghai University of Traditional Chinese Medicine. The patients/participants provided their written informed consent to participate in this study. Written informed consent was obtained from the individual(s) for the publication of any potentially identifiable images or data included in this article.

## Author contributions

LX and HW designed and drafted the manuscript. HH wrote the article and revised the manuscript. XC conducted the scale evaluation. YL and DH assisted in clinical treatment. All authors have read and agreed to the published version of the manuscript and contributed to the manuscript and approved the submitted version.

## Funding

This study was financially supported by the Scientific Research Project of Traditional Chinese Medicine Bureau of Guangdong Province (Grant No. 20222198).

## Conflict of interest

The authors declare that the research was conducted in the absence of any commercial or financial relationships that could be construed as a potential conflict of interest.

## Publisher's note

All claims expressed in this article are solely those of the authors and do not necessarily represent those of their affiliated organizations, or those of the publisher, the editors and the reviewers. Any product that may be evaluated in this article, or claim that may be made by its manufacturer, is not guaranteed or endorsed by the publisher.
